# Efficacy of Scintigraphy, Ultrasound and Both Scintigraphy and Ultrasonography in Preoperative Detection and Localization of Primary Hyperparathyroidism

**DOI:** 10.7759/cureus.4960

**Published:** 2019-06-20

**Authors:** Wafaa Abd Elhameed Elsayed, Rasha A Ali

**Affiliations:** 1 Nuclear Medicine, Sohag University Hospital, Sohag, EGY; 2 Epidemiology and Public Health, Sohag University Hospital, Sohag, EGY

**Keywords:** parathyroid scintigraphy, ultrasonography, primary hyperparathyroidism

## Abstract

Objective: The aim of our study was to evaluate the efficacy of preoperative dual-phase 99mTc-methoxyisobutylnitrile (MIBI) parathyroid scintigraphy (PS), and ultrasound (US) in primary hyperparathyroidism (pHPT) diagnosis and compare the results with the surgical findings.

Methods: Forty-five patients were enrolled in this study. Preoperative serum parathyroid hormone (PTH) levels, calcium (Ca), phosphate (P), and alkaline phosphatase (AP) levels were measured. All parathyroid patients were evaluated by ultrasonography, dual phase 99mTc-MIBI. Surgical findings were used as a reference standard.

Results: Of the 45 patients included in this study, 30 were females (66.7%) with an age range between 30 years and 70 years (mean age 41± 13). The sensitivity and specificity of 99mTc-MIBI scintigraphy was 97.4% and 71.4%, respectively, while the sensitivity of ultrasound was 94.4% and specificity 44.4%. The sensitivity, specificity, and accuracy of combined scintigraphy and ultrasound was higher-97.4%, 83.3%, and 95.6%, respectively.

Conclusions: The combination of MIBI and US appears promising for localizing parathyroid pathology in patients with primary hyperparathyroidism. The concordance rate is high together with a lower chance of missing concomitant thyroid pathology, which might alter the surgical approach.

## Introduction

Hyperparathyroidism is a generalized disorder of calcium (Ca), phosphate (P), and bone metabolism caused by increased secretion of parathyroid hormone (PTH). The elevation of PTH usually leads to hypercalcemia, hypophosphatemia, and relative hypocalciuria [1, 2].

Primary hyperparathyroidism may be caused by an adenoma or hyperplasia [3], secondary hyperparathyroidism is usually seen with renal failure and owing to various causes, such as osteomalacia, familial hypocalciuric hypercalcemia, and lithium therapy. Tertiary hyperparathyroidism occurs when one or more hyperplastic glands in secondary hyperparathyroidism begin functioning autonomously, resulting in hypercalcemia [2].

In the present study, we highlight the concern regarding primary hyperparathyroidism (PHPT) as its incidence is increasing with a rate of 42:100,000 per year and can reach up to 190:100,000 per year in women over 60 years of age [4].

The size of a normal pararthyroid gland ranges between 40-50 mg and therefore is infrequently visualized; however, when adenoma or hyperplasia occur the size increases up to ten fold and it can be detected in imaging modalities [5]. Since recommended by Coakley et al. in 1989, 99mTc-methoxyisobutylnitrile (MIBI) has gained great popularity in parathyroid imaging among the various subtraction methods such as 99mTc/201Tl, 123I/201Tl, 99mTc tetrofosmin/123I, and 99mTc-MIBI/123I [6-9]. 99mTc-MIBI is a radiopharmaceutical with lipophilic cationic properties, its distribution is initially proportional to blood ﬂow, and once intracellular, it is sequestered within mitochondria most actively in normal cardiac and thyroid cells [10, 11]. The uptake is especially prominent in overactive parathyroid glands, and maximum activity in thyroid gland is reached within five minutes, whereas parathyroid activity is sustained and washout is delayed, allowing the acquisition of the parathyroid glands nearly two hours after injection [12].

In recent years, minimally invasive parathyroidectomy has challenged the traditional bilateral neck exploration for PHPT, with advantages of reducing the rate of early postoperative hypocalcemia, less postoperative pain, and smaller scar [13]. However, this new approach needs a better preoperative imaging to help in localizing the diseased parathyroid gland to help in increasing the success rate of this less invasive surgery. Surgeons and endocrinologists keenly request information about the presence and precise location of the parathyroid glands.

Thus, the aim of the current study was to assess the efficacy of both dual-phase 99mTc-MIBI and US as diagnostic imaging tools for the localization of enlarged parathyroid glands in patients admitted to our department and prepared for parathyroidectomy and compare the results with the surgical findings.

## Materials and methods

Research design

The current study is a retrospective study where US and SC examinations data of 45 operated patients with clinically and biochemically documented 1ry hyperparathyroidism were examined and analyzed. The diagnosis of 1ry hyperparathyroidism was determined based on parathyroid hormone levels, serum calcium and phosphate, in addition to clinical evaluation of the patients, which included taking medical history, physical examination, laboratory tests, neck ultrasonography and 99mTc-MIBI scintigraphy of the neck and upper mediastinum.

Scintigraphy

All patients underwent a 99mTc-hexakis-2-methoxyisobutylisonitrile (99mTc-MIBI) scintigraphy with the use of a gamma camera. LEHR (low energy high resolution) collimators were used. Images were registered with a 128 × 128 matrix. The 99mTc-MIBI 20-25 mci was injected intravenously; early dynamic images and planar images were obtained 10 minutes after injection, and this permitted diagnosis by observing the differential washout from the thyroid and hyperfuncioning parathyroid. The “late” neck acquisition was performed 120 minutes after 99mTc-MIBI injection. In delayed images, much of the thyroid uptake has washout and the typical finding of hyperfunctioning parathyroid is a focus of residual activity in the neck with a high target to background ratio. Multiple hyperplastic glands may be seen, or in some patients the washout rate of the thyroid and hyperfunctioning parathyroid will be similar. However, often in these cases, the parathyroid adenoma can be seen as a distinct focus with a background of thyroid activity. Although adenomas are commonly detected contiguous to the thyroid or occasionally intrathyroidal, they may be ectopic, anywhere from high in the neck down to the mediastinum.

Ultrasound Examination

It was used to assess the location of the parathyroid glands, their size, and echo structure. Cross-sectional and longitudinal images of the anterior region of the neck were also obtained. The bilateral assessment was performed in the region from the common carotid artery bifurcation to the midline and down to the sternum. An attempt to visualize the superior part of the mediastinum was performed using GE Logiq P9 medical system (GE Healthcare, Chicago, IL, USA) using the superficial transducer ML-15.

Ethical considerations

This study complies with the regional and institutional ethical guidelines and with the Declaration of Helsinki. A written informed consent was obtained from the patients or his/her relatives to participate in the study.

## Results

Forty-five patients were included in this study, and 30 were females (66.7%) and 15 were males (33.3%), with an age range between 30 years and 70 years (mean age 41± 13).

The results of ultrasonography and 99mTc-MIBI scintigraphy examination of 45 patients were reviewed and analyzed. 99mTc-MIBI scintigraphy data was true positive in 37, false positive in two, true negative in five, and false negative in one case. The sensitivity and specificity of 99mTc-MIBI scintigraphy was 97.4% and 71.4%, respectively. Ultrasound results were true positive in 34, false positive in five, true negative in four and false negative in two cases. The sensitivity of ultrasound was 94.4% and specificity was 44.4%.

However, ultrasonography and scintigraphy combined showed higher sensitivity, specificity, and accuracy as shown in Tables 1-2.

**Table 1:** Shows true positive, true negative, false positive, false negative values for ultrasonography, 99mTc-MIBI scintigraphy, and both combined. US - ultrasound, MIBI - methoxyisobutylnitrile

	True positive	True negative	False positive	False negative	Total
US	34	4	5	2	45
MIBI	37	5	2	1	45
Combined US and MIBI	38	5	1	1	45

**Table 2:** Shows sensitivity, specificity, accuracy, positive and negative predictive values for ultrasonography, 99mTc-MIBI scintigraphy, and both combined. US - ultrasound, MIBI - methoxyisobutylnitrile

	Sensitivity (%)	Specificity (%)	Accuracy (%)	Positive predictive value (%)	Negative predictive value (%)
US	94.4%	44.4%	84.4%	87. 2%	66.7%
MIBI	97.4%	71.4%	93.3%	94.9%	83.3%
Combined US and MIBI	97.4%	83.3%	95.6%	97.4%	83.3%

## Discussion

Parathyroid hormone is the main regulator of calcium homeostasis in the human body. Primary hyperparathyroidism (PHPT) results from the inappropriate overproduction of parathyroid hormone from one or many parathyroid gland(s). It is the third most common endocrine disorder affecting 0.3% of the general population [14-16].

The main objective of our current study was to assess the efficacy of preoperative parathyroid scintigraphy, ultrasound, and both ultrasonography and parathyroid scintigraphy combined in primary hyperparathyroidism. Knowing the more correct preoperative diagnostic imaging technique is vital for better operative and postoperative outcomes.

A meta-analysis performed by Ruda et al. [17] encompassing 54 studies done between 1995 and 2003 using ultrasonography for preoperative localization in primary hyperparathyroidism calculated ultrasonographic sensitivity for the detection of adenoma to be 79%. Other studies reported preoperative ultrasonography sensitivities for the detection of solitary parathyroid adenomas to range from 72% to 89% in large series [18,19].

In agreement with an earlier study by Ibrahim and Elsadawy [20], it was shown in the current study that the US has a 94.6% sensitivity. Actually, ultrasonographic sensitivity could be related to the experience of the radiologist, which could explain the variability.

**Figure 1:**
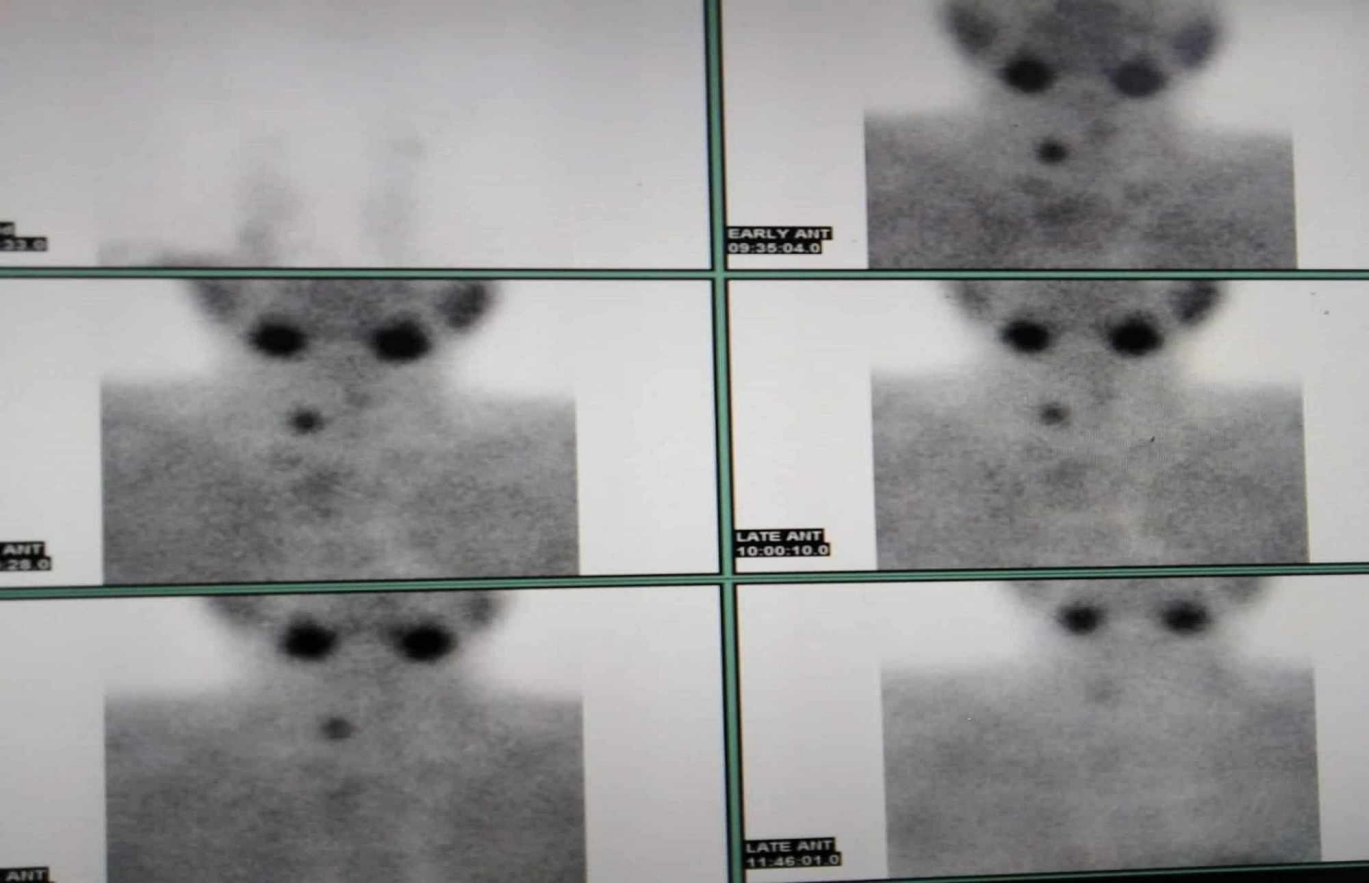
A female patient who complained of bony pains and had a parathormone level of 1045 underwent 99mTc-MIBI parathyroid scintigraphy that showed a rounded area of increased parathyroid activity at the lower right neck (early images) and persisted, becoming more obvious in time, denoting parathyroid adenoma.

99mTc-MIBI is the most frequently used radiotracer for imaging the parathyroid glands; it is taken up by both the thyroid and parathyroid glands, but adenomatous and hyperplastic parathyroid tissue displays more avid uptake of the radiotracer and frequently retains the radiotracer longer than adjacent thyroid tissue (Figure 1). The image shows the 99mTc-MIBI parathyroid scintigraphy of a female patient who complained of bony pains and had a parathormone level of 1045. A rounded area of increased parathyroid activity at the lower right neck visible in the early images persisted to become more obvious in time, denoting parathyroid adenoma.

Thus, initial planar images obtained shortly after the administration of radiotracer will show both thyroid and parathyroid tissue [12, 21]. Asymmetric foci of increased radiotracer uptake on early images can be perceived, representing abnormal parathyroid tissue superimposed on the normal thyroid. Delayed images hours after radiotracer administration are acquired to look for foci of retained radiotracer characteristic of hyperfunctioning parathyroid tissue [14-16].

In the current study, combined scintigraphy and ultrasound had a higher sensitivity, specificity, and accuracy than either technique alone, which is similar to the results of previous studies that noted improved accuracy, overall specificity, and positive predictive values when both techniques are combined preoperatively [5,8,22-24].

In contrast to other studies that suggest similar sensitivities and specificities for solitary adenoma detection [25], Lumachi et al. (1999) reviewed preoperative sonography and 99mTc-sestamibi findings in patients with proven solitary adenomas and found a combined sensitivity of 95% versus 80% for sonography and 87% for scintigraphy alone.

Actually, MIBI provides guidance for the interpretation of US data, especially in the case of ectopic parathyroid glands, small parathyroid adenomas, and concurrent thyroid nodules. US offers detailed anatomic information. It can also be especially helpful in patients with more than one enlarged parathyroid gland. In the majority of cases, the dual utilization of MIBI and US was able to successfully overcome the inherent limitations of each modality when employed alone. This is especially important in patients living in endemic goiter areas and it is important to screen nodular lesions of the thyroid gland as the leading cause of false positive results in both methods.

## Conclusions

The combination of scintigraphy and ultrasound techniques is highly efficient for localizing an enlarged parathyroid gland, and the chance of missing concomitant thyroid pathology is lower. In addition, the combination of both techniques is highly recommended if the surgeon is planning to perform unilateral neck exploration or minimally invasive surgery.
